# Survival outcomes and relapse patterns in high-risk metastatic neuroblastoma treated with radiotherapy-only consolidation in a resource-limited setting: a retrospective study from a lower-middle-income country

**DOI:** 10.3389/fonc.2026.1686737

**Published:** 2026-03-12

**Authors:** Haydar Hamza Alabedi, Noor Muhammed Abdulrahman, Ali Hatem Al-Rudayni

**Affiliations:** 1 College of Medicine, University of Baghdad, Baghdad, Iraq; 2 Scientific Department, Warith International Cancer Institute, Karbala, Iraq; 3 Department of Pediatric Oncology, Pediatric Teaching Hospital, Kirkuk, Iraq

**Keywords:** high-risk metastatic neuroblastoma, LMIC (low- and middle-income countries), radiotherapy, relapse, survival

## Abstract

**Background:**

High-risk metastatic neuroblastoma (HR-MNB) requires intensive multimodal therapy for durable remission. In many low- and middle-income countries (LMICs), access to autologous stem cell transplantation (ASCT) and maintenance immunotherapy remains limited. This study evaluated survival outcomes and relapse patterns among patients with HR-MNB treated with radiotherapy-only consolidation after achieving a complete or near-complete response to induction chemotherapy in Iraq.

**Methods:**

We conducted a retrospective cohort study of children with HR-MNB treated between December 2023 and March 2024 at a national radiotherapy center. Eligible patients received standard induction chemotherapy and consolidation radiotherapy with curative intent, without ASCT. Relapse-free survival (RFS) and overall survival (OS) were estimated using Kaplan–Meier methods. Relapse patterns were classified as local, distant, new-site, or combined failures. Subgroup analyses examined associations between survival outcomes and radiotherapy dose, age, and metastasis sites.

**Results:**

Twenty-five patients (median age, 48 months) were included. The median RFS from diagnosis was 18 months (95% confidence interval (CI), 14.4–21.6), and the median OS was 20.1 months (95% CI, 16.2–23.9). From radiotherapy initiation, the median RFS and OS were 5.8 and 11.4 months, respectively. Relapse occurred in 84% of patients, most frequently at new anatomical sites (56%). Subgroup analyses revealed no significant differences in survival based on radiotherapy dose or baseline characteristics.

**Conclusions:**

Radiotherapy-only consolidation following induction chemotherapy provided limited systemic disease control, reflected in early relapses and modest survival. These findings highlight the urgent need to expand access to ASCT, immunotherapy, and integrated care pathways to improve outcomes for HR-MNB in LMICs. Reporting real-world data from resource-constrained settings contributes essential evidence to guide equitable neuroblastoma treatment strategies globally.

## 1 Introduction

Neuroblastoma is the most common extracranial solid tumor in childhood and arises from the neural crest elements of the sympathetic nervous system. It primarily affects infants and young children and is characterized by substantial clinical and biological heterogeneity. Prognosis varies considerably according to the age at diagnosis, stage, histopathological features, and molecular markers, including MYCN amplification and segmental chromosomal aberrations (1).

Globally, an estimated 5,560 new cases of neuroblastoma occur annually in children aged <15 years, resulting in approximately 1,977 deaths. In the Middle East and North Africa region, where comprehensive pediatric cancer registries are limited, neuroblastoma remains underreported. However, regional estimates for 2021 indicate approximately 247 incident cases and 354 deaths (2, 3).

The Children’s Oncology Group (COG) and the International Society of Pediatric Oncology European Neuroblastoma Group (SIOPEN) classify neuroblastoma into low-, intermediate-, and high-risk categories based on age, International Neuroblastoma Risk Group staging, histology, MYCN amplification, DNA ploidy, and segmental chromosomal aberrations. High-risk neuroblastoma (HR-NB) is frequently metastatic at diagnosis, with common sites including the bone marrow, bone, liver, and lymph nodes, and is associated with poor overall survival (OS) (4, 5).

The current standard of care for HR-NB includes induction chemotherapy to achieve maximal tumor reduction followed by consolidation with high-dose chemotherapy and autologous stem cell transplantation (ASCT). Data from the ANBL0532 randomized trial demonstrated superior event-free survival (EFS) with tandem versus single ASCT, leading to the current COG recommendation in favor of tandem ASCT for most patients with HR-NB (6, 7). The SIOPEN HR-NBL1 protocol favors single ASCT with busulfan–melphalan as the preferred conditioning regimen, based on phase 3 evidence supporting its efficacy (8).

Radiotherapy plays a central role in the consolidation phase by eradicating the residual disease at the primary tumor site and metastatic foci in selected cases. Despite surgical resection and intensive chemotherapy, the risk of microscopic residual disease remains high, necessitating the routine use of consolidative radiotherapy, regardless of surgical completeness (9, 10). Radiation doses ranging from 21 to 36 Gy are typically administered and tailored according to the extent of resection, residual disease burden, and surrounding organ sensitivity (8).

Radiotherapy is frequently used as the sole form of consolidation in resource-limited settings where access to ASCT is limited. Although this approach cannot replace the systemic disease control provided by ASCT, followed by a maintenance regimen containing anti-GD2 immunotherapy, it may still provide meaningful local control, particularly in patients who achieve a complete or near-complete response to induction chemotherapy. Nevertheless, long-term survival in this context remains suboptimal because of the high risk of systemic relapse (11–13).

This retrospective study evaluated the relapse-free survival (RFS), OS, and recurrence patterns in patients with high-risk metastatic neuroblastoma (HR-MNB) who achieved a complete or near-complete response to induction chemotherapy and subsequently received radiotherapy as the sole consolidation modality. By emphasizing outcomes in a context where ASCT is not yet widely available, we aimed to highlight the importance of equitable access to comprehensive neuroblastoma treatment options.

## 2 Materials and methods

### 2.1 Study design and setting

This retrospective cohort study assessed RFS, OS, and recurrence patterns in patients with HR-MNB who achieved a complete or near-complete response to induction chemotherapy and received consolidation radiotherapy. This study was conducted at the Baghdad Radiotherapy and Nuclear Medicine Center located within the Baghdad Medical City Complex in Iraq. The data were collected between December 2023 and March 2024.

### 2.2 Ethics approval

The Institutional Review Board of the Department of Surgery, College of Medicine, University of Baghdad approved this study (Approval No. 1674; December 26, 2023). All data were collected and analyzed in accordance with the ethical standards and institutional guidelines.

### 2.3 Eligibility criteria

Patients with confirmed HR-MNB who received standard induction chemotherapy (with or without surgery), achieved complete or near-complete response post-induction, and received consolidation radiotherapy with curative intent were included in this study.

Patients who received palliative radiotherapy, had an unfavorable response to induction therapy, did not undergo radiotherapy, had incomplete data, or were lost to follow-up were excluded from the study.

### 2.4 Data collection

Data were extracted from paper-based medical records. Variables included demographic information such as sex, age, and diagnosis date; clinical information such as tumor location and metastatic sites; and treatment-related information such as chemotherapy regimen, response, surgery status, and radiotherapy site/dose/fractionations. Outcomes included relapse site and date, survival, and last follow-up date (mortality status).

Data entries were verified for consistency. Only patients treated with curative-intent radiotherapy were included.

### 2.5 Statistical analysis

Descriptive statistics were used to summarize baseline characteristics. OS and RFS were estimated using the Kaplan–Meier method with 95% confidence intervals (CIs). RFS was calculated both from the date of diagnosis and from the initiation of consolidation radiotherapy to reflect systemic and post-radiotherapy disease control. Differences between survival curves across predefined subgroups (radiotherapy dose, age category, and metastatic site) were compared using the log-rank test. Patients who remained alive without relapse were censored at the date of the last follow-up or, if unavailable, at the study end date. Median follow-up duration was estimated using the reverse Kaplan–Meier method. Kaplan–Meier curves are presented with accompanying number-at-risk tables and censoring marks to enhance interpretability. Statistical significance was defined as p < 0.05. All analyses were performed using IBM SPSS Statistics for Windows, version 25.0 (IBM Corp., Armonk, NY, USA).

### 2.6 Radiotherapy technique and treatment planning

Radiotherapy was delivered using computed tomography (CT)-based simulation and conformal treatment planning. All patients underwent immobilization using a customized thermoplastic mask, followed by simulation on a dedicated CT simulator with a slice thickness of 2.5–3 mm. Target volumes were contoured according to standard pediatric radiotherapy guidelines. The gross tumor volume represented the post-induction, pre-operative residual disease observed via CT imaging. A clinical target volume margin of 1.0–1.5 cm was added to account for potential microscopic extension, followed by a planning target volume expansion of 0.5–1.0 cm to accommodate daily setup uncertainties and patient motion.

Treatment planning was performed using 3D conformal radiotherapy on the XIO and MONACO systems. Beam arrangements typically consisted of multiple coplanar photon fields selected to optimize target coverage while respecting organ-at-risk constraints. The minimum field size used was 6 cm. Radiation was delivered using a medical linear accelerator with megavoltage photon beams. Dose prescription followed institutional protocols based on age, disease status, and residual tumor burden.

### 2.7 Relapse pattern classification

Relapse pattern classification was determined according to the COG/SIOPEN/MSKCC model and included local, distant, or new-site relapses. The site-specific model was classified as predefined (original sites) vs. new anatomical sites.

### 2.8 Limitations

The retrospective design, small sample size, and paper-based records may have introduced classification and selection biases. Despite this, the uniform treatment approach and data from a national referral center ensure a degree of internal consistency. These findings provide a novel perspective on previously undocumented LMIC settings.

## 3 Results

### 3.1 Patient characteristics

Overall, 25 patients with HR-MNB who achieved a complete response to induction chemotherapy and subsequently received consolidation radiotherapy with curative intent were included. The median age at diagnosis was 48 months (range, 20.4–84 months); 56% were male. The most common primary tumor site was the suprarenal region (84%), followed by the paravertebral (8%), carotid (4%), and mediastinal (4%) sites.

At the time of diagnosis, the most frequent site of metastasis was the bone marrow (48%), followed by bone (28%), brain (12%), and other locations (28%). All patients received systemic induction chemotherapy, and 80% underwent surgical resection. Consolidation radiotherapy was delivered to the primary tumor bed in all patients, with two (8%) also receiving radiotherapy for metastatic lesions (vertebrae and femur) **Table 1**).

**Table 1:** Baseline clinical and treatment characteristics of patients with high-risk metastatic neuroblastoma treated with consolidation radiotherapy after achieving complete or near-complete response to induction chemotherapy.

Characteristic	No. (%)
Sex	
Male	14 (56)
Female	11 (44)
Age (months)	
Median (range)	48 (20.4–84)
<60 months	17 (68)
≥60 months	8 (32)
Primary Tumor Site	
Suprarenal	21 (84)
Paravertebral	2 (8)
Carotid space	1 (4)
Mediastinal	1 (4)
Site of Metastasis	
Bone marrow	12 (48)
Bone	7 (28)
Brain	3 (12)
Other	7 (28)
Treatment Characteristics	
Induction Chemotherapy	25 (100)
Surgery	20 (80)
Consolidation Radiotherapy	25 (100)
Tumor bed treated	25 (100)
Secondary site irradiated	2 (8)
Femur	1 (4)
Vertebrae	1 (4)
Radiotherapy Dose (Gy/Fx)	
22 Gy/12 Fx	7 (28)
23 Gy/13 Fx	6 (24)
25.2 Gy/14 Fx	1 (4)
36 Gy/20 Fx	10 (40)
Unknown	1 (4)
Response to Treatment	
Complete response (CR)	23 (92)
Stable disease	2 (8)
Relapse Status	
No relapse	4 (16)
Relapse	21 (84)
Site of Relapse	
Primary tumor site	4 (16)
Distant relapse	6 (28)
New sites	14 (56)

RT, radiotherapy; Gy, Gray; Fx, fractions; CR, complete response.

### 3.2 Radiotherapy dose and classification

Radiotherapy dosing varied among the patients. Ten (40%) patients received high-dose radiotherapy (36 Gy in 20 fractions), whereas 14 (56%) received standard-intermediate doses ranging from 21 to 25.2 Gy. One patient received undocumented radiotherapy. For analytical purposes, the doses were grouped into the following two categories: standard intermediate (<36 Gy) and high (36 Gy) doses **Table 1**).

### 3.3 RFS

**Figure 1:**
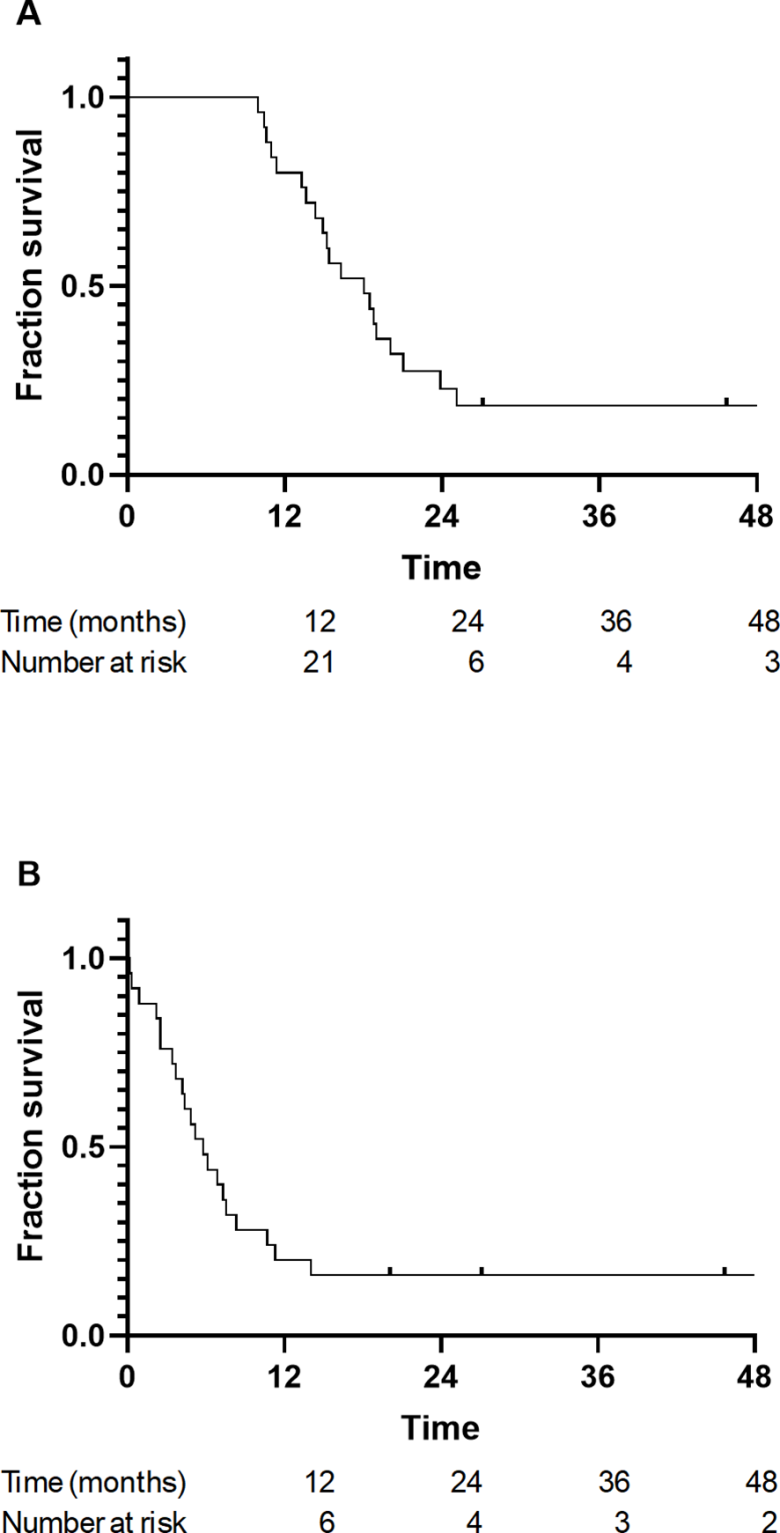
Kaplan–Meier curves for relapse-free survival (RFS) in patients with high-risk metastatic neuroblastoma treated with consolidation radiotherapy after achieving complete or near-complete response to induction chemotherapy. **(A)** RFS calculated from the date of diagnosis. **(B)** RFS calculated from the date of starting consolidation radiotherapy.

The median RFS from diagnosis was 18 months (95% confidence interval [CI], 14.4–21.6months) (**Figure 1A**). The median RFS from the start of radiotherapy was 5.8 months (95% CI, 2.55–7.45) (**Figure 1B**).

The Kaplan-Meier curves demonstrated a marked early decline in RFS, with the majority of relapse events occurring within the first year after radiotherapy. By 18 months post-radiotherapy, fewer than 20% of patients remained relapse-free. Stratified analyses based on radiotherapy dose (standard vs. escalated; p=0.95), age at diagnosis (>60months vs. <60months; p=0.57), and metastatic pattern (single site, multisite, and CNS involvement; p=0.81) at presentation did not reveal significant differences in RFS across subgroups (log-rank test, all p > 0.05).

### 3.4 Relapse patterns

During follow-up, disease relapse occurred in 21 of the 25 patients (84%). Relapse patterns were analyzed using both anatomical and international classification frameworks. Based on anatomical distribution, relapse at predefined sites (either the original primary tumor or known metastatic sites) was observed in 10 (48%) patients. Relapses at new sites (anatomical locations not involved in the initial diagnosis) occurred in 11 (52%) patients.

Relapses were further subclassified to align with the classification model employed by the COG, SIOPEN, and the Memorial Sloan Kettering Cancer Center. Local relapse only (primary tumor site) occurred in one (4%) patient, distant relapse only (initial metastatic sites) occurred in six patients (24%), new site relapse only occurred in 14 (56%), and combined relapse (involving >1 category, such as local + distant or distant + new) occurred in seven (28%).

### 3.5 OS

**Figure 2:**
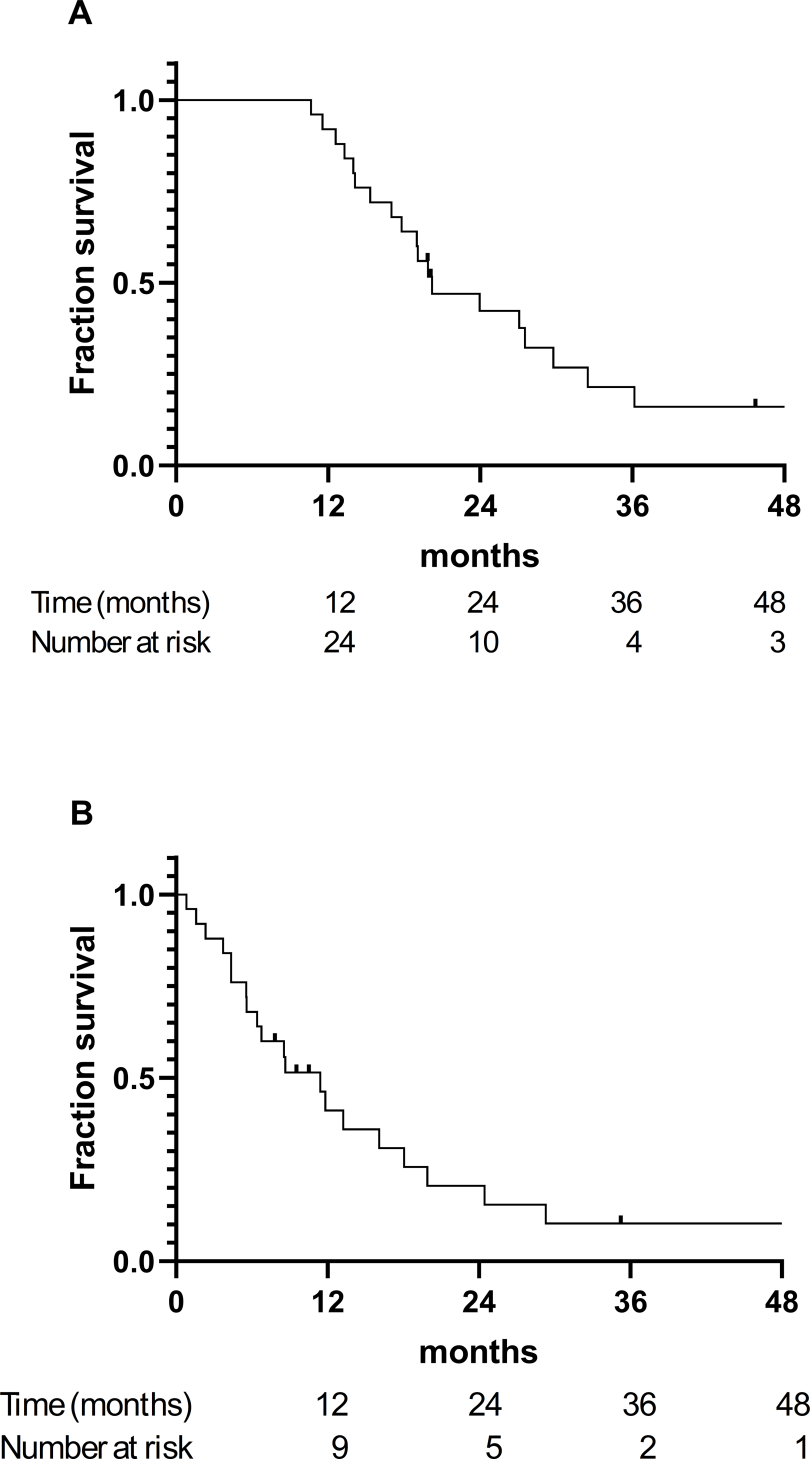
Kaplan–Meier curves for overall survival (OS) in patients with high-risk metastatic neuroblastoma treated with consolidation radiotherapy after achieving complete or near-complete response to induction chemotherapy. **(A)** OS calculated from the date of diagnosis. **(B)** OS calculated from the start of consolidation radiotherapy.

The median OS from diagnosis was 20.1 months (95% CI, 16.2–23.9; **Figure 2A**). The survival curve demonstrated a rapid decline during the first 18 to 24 months following diagnosis, after which a small plateau was observed, with fewer than 20% of patients remaining alive beyond 36 months. When measured from the start of radiotherapy, the median OS was 11.4 months (95% CI, 6.8–15.9; **Figure 2B**), reflecting the short interval between induction completion and subsequent relapse or death in this cohort. Censoring occurred infrequently, and the most deaths were observed early in follow-up, consistent with the aggressive clinical course of HR-MNB treated without ASCT or immunotherapy.

Subgroup analyses stratified by radiotherapy dose (standard vs. escalated; p=0.82), age at diagnosis (p=0.48), metastatic pattern (p=0.93), and relapse pattern (p=0.22), did not demonstrate statistically significant differences in OS (all p > 0.05). The survival curves for each subgroup showed broadly overlapping trajectories, indicating that no examined clinical or treatment characteristic meaningfully influenced OS in this radiotherapy-only consolidation cohort.

## 4 Discussion

This study evaluated survival outcomes and relapse patterns in patients with HR-MNB treated with consolidation radiotherapy as the sole post-induction modality following a complete or near-complete response to chemotherapy. Conducted in a resource-limited setting, this retrospective analysis provides real-world insights from a country with limited access to standard-of-care consolidation such as ASCT and maintenance modalities, including anti-GD2 immunotherapy.

The survival outcomes in our cohort were notably inferior to those reported for standard multimodal regimens. Median OS and RFS were 20.1 and 18 months, respectively, from diagnosis. Post-radiotherapy OS and RFS were as low as 11.4 and 5.8 months, respectively. These outcomes underscore the limited durability of radiotherapy as a stand-alone consolidation approach for HR-MNB. In contrast, in high-income countries, integrating ASCT followed by a maintenance regimen containing anti-GD2 monoclonal antibodies and isotretinoin into post-induction therapy has significantly improved long-term outcomes. For example, the ANBL0032 trial demonstrated that patients receiving this comprehensive consolidation strategy had a 5-year OS exceeding 60% (14). Similarly, the HR-NBL1/SIOPEN phase 3 trial, which used a single ASCT, reported a 3-year OS and EFS of 60% and 50%, respectively (8). Tandem ASCT, currently endorsed by the COG, has been shown to further improve EFS over single-transplant approaches (6).

Our findings align more closely with those observed in LMICs, where access to optimal systemic consolidation and maintenance modalities is constrained. As reported by van Heerden and Kruger, the absence of ASCT and anti-GD2 immunotherapy in LMICs is associated with poorer survival outcomes than those achieved in high-income settings (13). An Indian cohort treated with a non-myeloablative TVD-based regimen (topotecan, vincristine, and doxorubicin) and radiotherapy, but without ASCT or immunotherapy, achieved a 4-year EFS of 29.3% (15). While inferior to outcomes achieved with ASCT or anti-GD2 immunotherapy protocols, these results exceeded those observed with radiotherapy alone, highlighting the critical role of systemic therapy in prolonging remission and survival (8, 14). Although direct comparison across studies should be approached with caution, given differences in patient demographics, treatment infrastructure, and supportive care, the findings by Jain et al. offer a relevant point of reference for evaluating alternative consolidation approaches in resource-constrained settings. Their experience underscores the potential role of intensive chemotherapy consolidation in the absence of ASCT. Building on this evidence, further studies are needed to guide the development of context-adapted treatment strategies that balance efficacy and feasibility in LMICs.

Our findings support the consideration of interim consolidation strategies using accessible chemotherapeutic agents in contexts where ASCT is not feasible. In our cohort, it was unclear why chemotherapy-based consolidation regimens were not employed, although fragmented documentation and limited post-treatment follow-up constrained our ability to explore this further. Subgroup analyses stratified based on age, metastasis site, and radiotherapy dose yielded no statistically significant differences in OS or RFS. These uniformly poor outcomes across the strata emphasize the limitations of radiotherapy-only consolidation and reflect the modest statistical power of our sample size (n = 25).

In our retrospective analysis, 84% of the patients experienced relapse, most commonly at new anatomical sites (56%), followed by distant relapse at the initial metastatic sites (24%) and local recurrence at the primary tumor site (4%). Combined patterns involving multiple relapse categories were observed in 28% of the cases. These dynamics align with the relapse patterns reported by the COG, SIOPEN, and the Memorial Sloan Kettering Cancer Center, where systemic and new-site failures remain predominant despite favorable local control rates. This reinforces the principle that effective post-remission therapy targets systemic micro metastatic diseases and not solely the primary tumor site.

The relapse patterns and survival outcomes observed in our cohort are best interpreted in the context of prior cooperative group analyses that evaluated radiotherapy distribution as part of intensive multimodal consolidation strategies. These studies provide insights into how differences in radiotherapy exposure influence relapse patterns, without demonstrating a consistent survival benefit. Li et al. showed that the total body irradiation used during transplant conditioning was associated with a lower risk of recurrence at previously involved metastatic sites (16). However, relapse frequently occurred at new distant sites, and no improvement in RFS was observed, indicating that broader radiation exposure did not result in durable disease control (16). Similarly, Polishchuk et al. reported that skeletal relapses in high-risk neuroblastoma most often occurred at bone sites involved at diagnosis (17). Although irradiated bone lesions showed fewer recurrences than unirradiated sites, this approach did not alter overall relapse patterns (17). Two patients in our cohort received consolidative radiotherapy to bone metastatic sites in addition to primary-site irradiation after achieving a complete response to induction chemotherapy. In one case, the patient remained alive and relapse-free until the end of follow-up. In the other case, the patient experienced systemic relapse and subsequently died. The decision to irradiate bone metastatic sites in these two patients was individualized and not part of the standard treatment protocol used during the study period.

Contemporary COG studies have shown that local control after primary site consolidation radiotherapy is generally favorable. In the A3973 trial, Braunstein et al. found that prophylactic irradiation of uninvolved regional lymph node stations did not significantly improve local progression, event free survival, or OS (18). These findings support the strategy of limiting radiotherapy fields to the primary tumor bed and involved nodal regions. Similarly, in the ANBL0532 trial, escalation of the radiotherapy dose to the primary site to 36 Gy for gross residual disease did not significantly improve local control, RFS, or OS compared with standard dose radiotherapy (19). This finding parallels our internal subgroup analysis, in which survival outcomes did not significantly improve (OS, p=0.82 and RFS, p=0.95) when comparing the escalated dose of 36 Gy to the standard intermediate dose of 20–25 Gy. Together, these results indicate that standard primary site consolidation radiotherapy achieves low rates of local progression and that further increases in dose or field size do not provide additional survival benefit in high-risk neuroblastoma.

In this context, the limited survival outcomes observed in our cohort, treated with radiotherapy-only consolidation in the absence of ASCT or anti GD2 therapy, highlight the central importance of systemic consolidation rather than further intensification or expansion of radiotherapy for achieving durable remission in high-risk metastatic neuroblastoma.

Our study reflects broader challenges that are likely relevant to other LMICs. Within the study period, risk stratification was performed solely on factors such as age and stage of disease, while MYCN amplification testing, which is a cornerstone of neuroblastoma risk stratification, was not performed. Because MYCN amplification is a central component of the International Neuroblastoma Risk Group and COG risk-classification systems, the absence of this modality likely resulted in under-recognition of biologically aggressive disease. We emphasize this as a key systemic limitation during the study period, which is now partially being addressed as molecular diagnostics are gradually becoming available in Iraq. Additionally, salvage therapies after relapse could not be analyzed due to frequent external care and incomplete documentation specially in context of rapid clinical deterioration. As a result, salvage therapies were not reported.

Our study reflects the broader systemic challenges faced by pediatric oncology programs in Iraq and is likely relevant to other LMICs. During the study period, MYCN amplification testing, which is a cornerstone of neuroblastoma risk stratification, was not performed. Anti-GD2 immunotherapy was not accessible through the national formulary, and documentation on the use of isotretinoin for maintenance therapy was inconsistent. Radiotherapy is frequently delayed, with median intervals exceeding 6 months from diagnosis, reflecting limited infrastructure, fragmented referral pathways, and the absence of integrated, timely treatment coordination. Furthermore, owing to scattered and incomplete documentation, it is difficult to assess the quality and consistency of supportive care measures, which are important determinants of outcomes in HR-NB. We included this observation for transparency because these limitations may have influenced the observed survival and relapse outcomes. Additionally, structured post-treatment surveillance and digital patient tracking systems are lacking, hindering the early detection of relapse and long-term outcome monitoring. Public awareness of the importance of completing all treatment phases, including consolidation and maintenance, remains limited. These systemic gaps mirror those described in other LMIC contexts, including sub-Saharan Africa and the Middle East, where delayed radiotherapy, poor integration of services, and treatment abandonment are common (12, 13, 16, 17, 20, 21). Notably, Iraq has recently made significant progress in expanding its ASCT capacity across public and private hospitals. This development may reduce the burden of cross-border treatment and represent a vital step toward aligning national practices with international standards.

This study had some limitations. The small sample size reduces the power required to detect meaningful subgroup differences and limits generalizability. As this was a single-center retrospective study, the findings were subject to selection and information bias, particularly due to the reliance on handwritten medical records. Nonetheless, the cohort’s clinical homogeneity and treatment within a centralized national cancer center conferred internal consistency and underscored the need for broader prospective data collection in the region.

In conclusion, in this retrospective analysis, radiotherapy-alone consolidation therapy following a complete or near-complete response to induction chemotherapy offered modest local control but did not sufficiently prevent systemic relapse in children with HR-MNB treated in a resource-limited setting. These findings emphasize the significance of multimodal consolidation, including ASCT, in attaining a durable remission. Our results reflect the real-world limitations of care delivery in LMICs and underscore the urgent need to expand access to comprehensive risk-adapted treatment strategies. By documenting survival outcomes and relapse patterns in an underrepresented LMIC context, this study contributes to the growing call for the equitable implementation of evidence-based protocols and capacity-building initiatives in global pediatric oncology.

## Data Availability

The raw data supporting the conclusions of this article will be made available by the authors, without undue reservation.

## Supplementary material

Supplementary Table 1 Subgroup analyses of overall survival (OS) and relapse-free survival (RFS) according to radiotherapy dose, age at diagnosis, and metastatic pattern at diagnosis in patients with high-risk metastatic neuroblastoma. Median survival estimates were calculated using the Kaplan–Meier method. P-values were derived from log-rank tests comparing subgroups within each variable; for metastatic pattern, the p-value reflects an overall comparison across all listed categories. One patient was excluded from the radiotherapy dose–based analysis because radiotherapy dose information was unavailable. Age categories were defined pragmatically based on cohort distribution, as no patients were diagnosed before 18 months of age. Subgroup analyses were exploratory and should be interpreted with caution given the small sample size.
