# Supplementary material for: Survival outcomes and relapse patterns in high-risk metastatic neuroblastoma treated with radiotherapy-only consolidation in a resource-limited setting: a retrospective study from a lower-middle-income country
Source: Front Oncol. 2026 Mar 12;16:1686737. doi: 10.3389/fonc.2026.1686737 (PMC13017277; doi:10.3389/fonc.2026.1686737)
Supplement: Supplementary Table 1 — Subgroup analyses of overall survival (OS) and relapse-free survival (RFS) according to radiotherapy dose, age at diagnosis, and metastatic pattern at diagnosis in patients with high-risk metastatic neuroblastoma. Median survival estimates were calculated using the Kaplan–Meier method. P-values were derived from log-rank tests comparing subgroups within each variable; for metastatic pattern, the p-value reflects an overall comparison across all listed categories. One patient was excluded from the radiotherapy dose–based analysis because radiotherapy dose information was unavailable. Age categories were defined pragmatically based on cohort distribution, as no patients were diagnosed before 18 months of age. Subgroup analyses were exploratory and should be interpreted with caution given the small sample size. [file Table1.docx]

**Supplementary Table S1.** Subgroup analyses of overall survival (OS) and relapse-free survival (RFS) according to radiotherapy dose, age at diagnosis, and metastatic pattern at diagnosis in patients with high-risk metastatic neuroblastoma. Median survival estimates were calculated using the Kaplan–Meier method. P-values were derived from log-rank tests comparing subgroups within each variable; for metastatic pattern, the p-value reflects an overall comparison across all listed categories. One patient was excluded from the radiotherapy dose–based analysis because radiotherapy dose information was unavailable. Age categories were defined pragmatically based on cohort distribution, as no patients were diagnosed before 18 months of age. Subgroup analyses were exploratory and should be interpreted with caution given the small sample size.

| **Subgroup Variable** | **Category** | **N** | **Median OS (months)** | **p-value (OS)** | **Median RFS (months)** | **p-value (RFS)** |
| --- | --- | --- | --- | --- | --- | --- |
| **Radiotherapy dose** | Standard dose (<36 Gy) | 14 | 20 | **0.82** | 15.4 | 0.95 |
|  | Escalated dose (36 Gy) | 10 | 19 |  | 18 |  |
| **Age at diagnosis** | <60 months | 17 | 20 | **0.48** | 16.3 | 0.58 |
|  | ≥60 months | 8 | 19 |  | 18 |  |
| **Metastatic pattern at diagnosis** | Single site | 15 | 20 | **0.93** | 15.4 | 0.81 |
|  | Multisite metastases | 7 | 20 |  | 18.4 |  |
|  | CNS involvement | 3 | 28 |  | 18 |  |
